# Prognostic factors and sites of metastasis in unresectable locally advanced pancreatic cancer

**DOI:** 10.1002/cam4.459

**Published:** 2015-04-18

**Authors:** Renata D’Alpino Peixoto, Caroline Speers, Colleen E McGahan, Daniel J Renouf, David F Schaeffer, Hagen F Kennecke

**Affiliations:** 1 Division of Medical Oncology, University of British Columbia, British Columbia Cancer AgencyVancouver, British Columbia, Canada; 2 Pancreas CentreVancouver, British Columbia, Canada; 3 Gastrointestinal Cancer Outcomes Unit, British Columbia Cancer AgencyVancouver, British Columbia, Canada; 4 Cancer Surveillance & Outcomes, British Columbia Cancer AgencyVancouver, British Columbia, Canada; 5 Department of Pathology and Laboratory Medicine, University of British ColumbiaVancouver, British Columbia, Canada

**Keywords:** CA 19.9, LAPC, liver metastasis, locally advanced pancreatic cancer, metastatic sites, pancreatic ductal adenocarcinoma, PDAC, prognostic factors, survival

## Abstract

Due to differences in natural history and therapy, clinical trials of patients with advanced pancreatic cancer have recently been subdivided into unresectable locally advanced pancreatic cancer (LAPC) and metastatic disease. We aimed to evaluate prognostic factors in LAPC patients who were treated with first-line chemotherapy and describe patterns of disease progression. Patients with LAPC who initiated first-line palliative chemotherapy, 2001–2011 at the BC Cancer Agency were included. A retrospective chart review was conducted to identify clinicopathologic variables, treatment, and subsequent sites of metastasis. Kaplan–Meier and Cox-regression survival analyses were performed. A total of 244 patients were included in this study. For the majority of patients (94.3%), first-line therapy was single-agent gemcitabine. About 144 (59%) patients developed distant metastatic disease and the most frequent metastatic sites included peritoneum/omentum (42.3%), liver (41%), lungs (13.9%), and distant lymph nodes (9%). Median overall survival (OS) for the entire cohort was 11.7 months (95% CI, 10.6–12.8). Development of distant metastases was associated with significantly inferior survival (HR 3.56, 95% CI 2.57–4.93), as was ECOG 2/3 versus 0/1 (HR 1.69, 95% CI 1.28–2.23), CA 19.9 > 1000 versus ≤1000 (HR 1.59, 95% CI 1.19–2.14) and female gender, (HR 1.57, 95% CI 1.19–2.08). In this population-based study, 41% of LAPC patients treated with first-line chemotherapy died without evidence of distant metastases. Prognostic factors for LAPC were baseline performance status, elevated CA 19.9, gender, and development of distant metastasis. Results highlight the heterogeneity of LAPC and the importance of locoregional tumor control.

## Introduction

Pancreatic ductal adenocarcinoma (PDAC) is the fourth leading cause of cancer-related death in the United States 1. At the time of initial diagnosis, only 10–20% of the patients are candidates for surgery 2. An estimated 30–40% of patients present with unresectable locally advanced pancreatic cancer (LAPC) defined by any of the following: greater than 180° superior mesenteric artery or celiac encasement, aortic invasion, and unreconstructable superior mesenteric or portal vein involvement in the absence of metastatic disease 3. LAPC patients have a median overall survival (OS) of about 8–12 months 4–7. The remainder is diagnosed with metastases at presentation, with an estimated survival of 6 months when treated with single-agent gemcitabine 8.

Optimal treatment for LAPC remains controversial. Results of the phase III LAP 07 study have cast doubt on the role of external beam radiation therapy of the pancreatic bed in LAPC patients 9, but local control of disease and symptoms remains a major issue for such patients. Two recent phase III studies have demonstrated improved OS with FOLFIRINOX 10 and combination gemcitabine and nab-paclitaxel in the metastatic setting 11, but the benefit of these regimens in LAPC is unknown.

Locally advanced presentation may reflect a differing disease biology, which is corroborated by an autopsy series demonstrating that 30% of patients presenting with stage III (LAPC) disease succumbed to locally destructive disease without evidence of progression to distant sites 12. In contrast, patients with significant metastatic disease died more commonly as a consequence of either organ failure or cachexia 12.

The frequency with which LAPC patients develop visceral metastasis is not well described and may potentially influence therapeutic decisions. If the majority of LAPC patients eventually develop distant metastasis, this may imply that similar therapeutic agents may be relevant for both LAPC and metastatic PDAC. Conversely, if LAPC and metastatic PDAC have differing disease trajectories, this may imply that different treatment paradigms are relevant.

While LAPC and metastatic PDAC have historically been included together in phase III studies, recent consensus guidelines have suggested that the two groups be studied separately due to differences in natural history and therapy 13. Clinically validated prognostic factors are required for LAPC patients enrolled in clinical trials and may differ from those that are relevant for PDAC patients with more advanced disease. The presence of liver metastasis and the number of metastatic sites are significant prognostic factors among patients with metastatic pancreatic cancer 14, but these are not relevant considerations among patients with unresectable, nonmetastatic tumors.

The objectives of this study were to describe the pattern of disease progression including sites of metastatic disease among patients with unresectable LAPC treated with first-line chemotherapy and identify clinical prognostic factors in these patients.

## Material and Methods

### Patients and data sources

All patients with unresectable LAPC who initiated first-line palliative chemotherapy between 2001 and 2011 and were referred to one of five provincial British Columbia (BC) Cancer Agency clinics were included. The provincial pharmacy database was used to identify patients who had a pathological confirmation of PDAC and received at least once cycle of palliative-intent chemotherapy. Retrospective chart review confirmed that none of these patients had resection of their primary tumor at the time of initiation of therapy and that there was no evidence of distant metastasis. Of the 1042 charts reviewed, 736 were excluded from the study due to evidence of metastatic disease on staging, as determined by either positron emission tomography-computerized tomography (PET-CT) scan or CT scan prior to initiation of first-line chemotherapy. An additional 62 patients were excluded due to prior curative-intent resection. A total of 244 LAPC patients were included in the study.

Baseline demographics, tumor characteristics, treatment details, and outcomes were abstracted to an anonymized database and analyzed. Eastern Cooperative Oncology Group (ECOG) performance status (PS) was prospectively documented at the time of initial consultation and recorded in the BCCA Gastrointestinal Cancers Outcomes Unit (GICOU). ECOG status was inferred from retrospective chart review among cases where values were missing. Carbohydrate antigen (CA) 19.9 levels were obtained by chart review prior to onset of systemic therapy. All imaging reports from initiation of first-line chemotherapy to death or last follow-up were reviewed to investigate metastatic disease development. Sites of metastases were recorded from the first imaging report detected. Disease progression was determined by review of sequential CT scans. Patients were classified as “local only” if they never developed metastatic disease, based on both imaging and physical examination. The median time from last imaging to death or date of last contact was determined for patients with “local only” disease. Patients were classified as “peritoneum only” if their only site of metastatic disease was peritoneum/omentum/mesentery/ascites. Patients with intra-abdominal nodal metastasis, exclusively, were classified as “lymph nodes only”. Patients who developed any lung, liver, bone, or other distant metastasis were classified as “distant” disease. Those who developed both peritoneal and distant disease were defined as having “mixed” sites of metastases.

In multivariable analysis, cases with missing CA19.9 were excluded, and cases with missing information were considered to be randomly distributed. This study was approved by the local Institutional Review Board.

### Statistical analysis

Statistical analysis was performed using SPSS version 14.0 for Windows® (SPSS, Chicago, IL). OS was calculated in months from the time of primary diagnosis to date of death or last follow-up. Kaplan–Meier (KM) curves for OS were generated. The log-rank test was used to assess statistical differences among variables and *P* < 0.05 was considered statistically significant. Multivariable survival analyses were performed using Cox-proportional hazards models in order to explore the effect of variables on OS. Development of metastatic disease was analyzed as a time-dependent variable. Hazard ratios (HR) and 95% confidence intervals were calculated to estimate risk of death.

## Results

Median follow-up was 59.7 months. In this cohort, 244 patients with a median age of 65 years (range 36–88) presented with LAPC and began palliative chemotherapy. Patient and treatment characteristics are summarized in Table1. A total of 35 (14.3%) patients received locoregional radiation, with 11 patients receiving radiation as part of the initial treatment plan. The remaining 24 patients received radiation as a palliative measure, after completion of their initial treatment plan. None of the tumors were converted to a resectable status. Only 36 patients (14.8%) received second-line chemotherapy, 31 of whom had fluoropyrimidines (either 5-fluorouracil or capecitabine).

**Table 1:** Baseline and treatment characteristics of 244 LAPC patients

	Number	%
Age (range 36–88)	65 (median)	–
Gender		
Male	136	55.7
Female	108	44.3
Location		
Head	177	72.5
Body	56	23
Tail	11	4.5
Histology		
Ductal adenocarcinoma	232	95.1
Mucinous adenocarcinoma	9	3.7
Signet ring cell carcinoma	1	0.4
Adenosquamous carcinoma	1	0.4
Clear cell carcinoma	1	0.4
Clinical node status		
N0	186	76.2
N1	58	23.8
Ethnicity		
Caucasian	207	84.8
Asian	34	13.9
Other	3	1.2
Baseline ECOG		
0	12	4.9
1	134	54.9
2	73	29.9
3	25	10.2
Baseline CA19.9		
≥1000	73	30
<1000	150	61.4
Missing	21	8.6
First-line chemotherapy		
Gemcitabine alone	230	94.3
Gemcitabine combination	13	5.3
FOLFIRINOX	1	0.4
Pancreatic bed radiotherapy		
Yes	35	14
No	209	86
Subsequent surgical resection		
Yes	0	0
No	244	100

Number and percentage of patients in each category are shown.

Sequential CT reports were reviewed during time of chemotherapy and after progression until death or last follow-up. A total of 144 patients (59%) developed distant metastatic disease, with the sites of metastasis summarized in Table2. About 100 patients (41%) were classified as “local only” and had no evidence of metastatic disease by last follow-up or before their death. In the “local only” patients, median time from last imaging to death, or last follow-up was 1.9 months.

**Table 2:** Patterns of progression among 244 LAPC patients treated with first-line chemotherapy

	Number of patients	%
Development of metastatic disease		
Yes	144	59
No	100	41
Number of metastatic sites		
Single	116	80.5
Multiple	28	19.5

Sites of any metastasis	Single metastasis only *N* = 115 metastasis, 115 patients	Multiple metastatic sites *N* = 57 metastasis in 28 patients	Total metastasis to any site *N* = 172 metastasis in 144 patients
Peritoneum/omentum	46	15	61
Liver	40	19	59
Lungs	13	7	20
Distant lymph nodes	3	10	13
Bones	6	1	7
Spleen	3	3	6
Adrenals	2	1	3
Skin	1	0	1
Testicle	0	1	1
Pericardium	1	0	1

**Figure 1:**
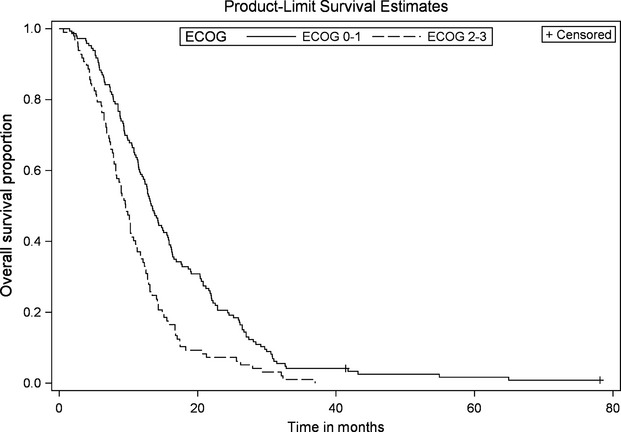
Overall survival by ECOG performance status. Kaplan–Meier curve of LAPC patients with ECOG PS 0–1 (*n* = 146) versus ECOG PS 2–3 (*n* = 98). *P *= 0.0002, HR 1.69 (95% CI 1.28–2.23).

**Figure 2:**
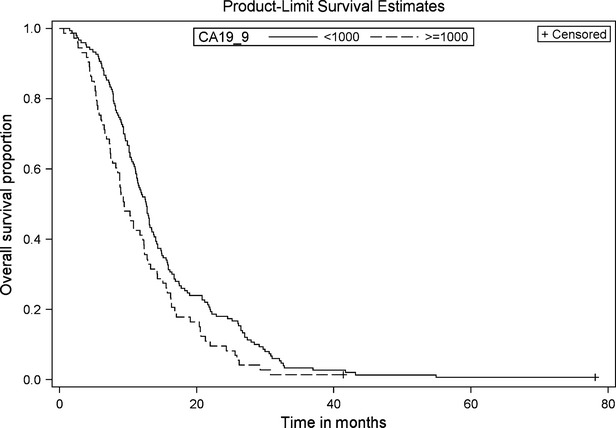
Overall survival by CA 19.9 levels. Kaplan–Meier curve of LAPC patients with CA 19.9 < 1000 (*n* = 150) versus CA 19.9 > 1000 (*n* = 73). *P*-value, 0.0018, HR 1.59 (95% CI 1.19–2.14).

OS for the entire cohort was 11.7 months (95% CI, 10.6–12.8). On univariate analysis, poor ECOG PS at diagnosis (PS ≥ 2), and high CA19-9 levels (≥1000) were significantly associated with worse OS (Figs.1 and 2). Patients with ECOG PS ≥ 2 had shorter median OS when compared to patients with PS of either 0 or 1 (9.5 vs. 13.3 months, *P* < 0.001, HR 1.79). The group of patients with high CA19-9 (≥1000) also had significantly shorter median OS as compared to the group with lower CA19-9 (9.4 vs. 12.6 months, *P* = 0.009, HR 1.46). Although there was a trend toward worse OS among females when compared to males (11.1 vs. 12.3 months, *P* = 0.093, HR 1.24), this did not reach statistical significance on univariate analysis. Age (≤65 vs. >65), location of the tumor (head vs. body/tail), and presence of locoregional lymph nodes on baseline CT scan were not significantly associated with inferior OS (*P* = 0.943, 0.479, and 0.427, respectively).

Multivariate analysis is shown in Table3, with the predictors of inferior OS were ECOG PS 2/3, elevated CA19-9, female gender, and development of metastatic disease (“peritoneal only”; “distant”; “mixed”).

**Table 3:** Multivariate analysis of prognostic factors for overall survival

Variables included in final model	*P*-value	Hazard ratio	Lower 95% CI	Upper 95% CI
Distant mets: yes (*n* = 144) versus no (*n* = 100)	<0.0001	3.56	2.57	4.93
Peritoneal (*n* = 61) versus no mets (*n* = 100)	<0.0001	4.30	2.97	6.24
Mixed mets (*n* = 28) versus no mets (*n* = 100)	<0.0001	3.64	2.00	6.63
ECOG: 2/3 (*n* = 98) versus 0/1 (*n* = 146)	0.0002	1.69	1.28	2.23
CA19-9: >1000 (*n* = 73) versus ≤1000 (*n* = 150)	0.0018	1.59	1.19	2.14
Sex: female (*n* = 108) versus male (*n* = 136)	0.0015	1.57	1.19	2.08

Mets, metastasis; ECOG, Eastern cooperative oncology group performance status.

## Discussion

The objectives of this study were to describe the pattern of disease progression in patients with unresectable LAPC treated with first-line chemotherapy including sites of metastatic disease, and to identify clinical prognostic factors in these patients. This study identified that baseline PS, CA 19-9 level, gender, and subsequent development of distant metastatic disease are all important prognostic factors in this population.

This study demonstrates that the majority of LAPC patients (59%) eventually developed metastatic spread and experience an inferior OS. The remaining 41% of the patients who present with LAPC succumb to the disease in the absence of distant peritoneal or other metastases. The findings highlight the heterogeneity of LAPC and importance of local tumor complications and control.

The median OS on all patients in this study was 11.7 months which compares favorably to reported survival durations for LAPC 4–7,15. Our finding, that an estimated 60% of patients develop distant metastatic disease, supports current NCCN and ESMO guidelines 16,17 suggesting a period of initial chemotherapy rather than immediate radiation for patients with unresectable LAPC to allow the determination of tumor responsiveness and asses the development of distant metastasis.

The frequency and distribution of metastatic spread described herein is similar to a previous analysis of an autopsy series of 76 patients with PDAC, in which only 18 had LAPC 12. Among them, 13 (72%) had evidence of metastatic disease at autopsy in addition to the locally advanced primary carcinoma, and five (28%) did not have any metastases at autopsy 12. This autopsy series also demonstrated that among all PDAC patients, the most common sites of metastases were liver (80%), peritoneum (48%), and lungs (45%). Our study showed lower rates of metastatic disease, which might be explained by the lower sensitivity of imaging for detection of a small focus of metastasis. In our study, peritoneum was the most common metastatic site (42.3%), then liver (41%), possibly reflecting a preferential spread by tumoral seeding in the presence of an advanced primary tumor. As expected, median OS was worse for patients who developed any sort of metastatic lesions when compared to those who did not. Development of peritoneal, distant metastases, or both were all associated with shorter OS when compared to local progression only.

The frequency of metastatic disease reported in patients enrolled on the LAP 07 study is strikingly similar to our findings 18. LAP 07 patients were initially randomized to 4 months of gemcitabine or gemcitabine plus erlotinib. Patients with nonprogressive disease (*n* = 269) were randomized to two additional months of chemotherapy or radiation with concurrent capecitabine. OS did not significantly differ between the treatment arms (15.2 vs. 16.5 months, *P* = 0.8). At the time of analysis, 238 patients developed progressive disease after the second randomization, which was locoregional in 96 (50.5%) and metastatic in 97 patients (49.5%). The findings are consistent with the frequency of metastasis described in the current study. OS was longer for patients enrolled in LAP 07 than described in the current study likely due to the exclusion of patients who progressed during the first 4 months of chemotherapy 18 and the population-based nature of the study. The authors of the LAP 07 study also reported the results of a multivariate analysis in which several baseline characteristics were investigated as prognostic factors for OS. Age (HR 1.01; 95% CI 1.00–1.03; *P* = 0.0418), pain (HR 1.36; 95% CI 1.08–1.71; *P* = 0.0094), albumin (HR 0.96; 95% CI 0.94–0.98; *P* = 0.0001) and tumor size (HR 1.01; 95% CI 1.00–1.02; *P* = 0.0033) were independent prognostic factors 19. The study also described prognostic factors in LAPC.

The value of CA 19.9 as a predictive and prognostic marker in metastatic PDAC is variable. An analysis of patients enrolled in a randomized trial of gemcitabine versus gemcitabine plus capecitabine indicated that the median OS for patients with a baseline CA 19-9 equal to or above the median value (i.e., 59× ULN) was 5.8 months (95% CI 5.1–7.0), which was significantly shorter than that for patients with baseline concentrations below the median value [10.3 months (95% CI 8.6–12.8), *P* < 0.0001] 20. A subsequent study in patients treated with combination nab-Paclitaxel and gemcitabine versus gemcitabine alone did not demonstrate the prognostic value of CA 19.9. Only Karnofsky PS (HR 1.56, 95% CI 1.29–1.888), presence or absence of liver metastasis (HR 1.79, 95% CI 1.32–2.42) and therapy were prognostic of survival 14. Elevated baseline CA 19.9 was reported as a significant prognostic factor in a study of 154 patients with advanced pancreatic cancer receiving chemotherapy (HR 1.8) 21, while another study reported an HR of 2.17 using a similar cut-point of less than, versus greater than 1000 22. Results of the current study support the importance of baseline CA 19.9 as an independent variable with a level above 1000 U/mL associated with a HR for death of 1.59 (*P* = 0.0018) in multivariate analysis.

The relevance of CA 19.9 in LAPC may differ from metastatic PDAC, where presence or absence of liver metastasis and number of metastatic sites may be more relevant determinants of OS. A subset of patients included in this study experienced obstructive jaundice and elevated serum bilirubin levels as a result of their LAPC. In the absence of malignancy, elevated serum bilirubin levels are documented to cause CA 19.9 elevations, therefore making CA 19.9 a less specific marker in this disease setting 23–26. However, the CA 19.9 elevation observed with nonmalignant hyperbilirubinemia are generally in the range of 2 times the upper limit of normal and significantly lower than those observed with pancreatic malignancy 24. The cutoff value for CA 19.9 elevation chosen in this study was over 25 times the upper limit of normal (>1000 IU), a level unlikely to ever be observed in a nonmalignant state. This use of this higher cut-point for defining CA 19.9 elevation may explain why this variable had such a significant prognostic impact in this study.

Performance status at initial diagnosis significantly influenced OS, consistent with the observations of other studies 21,22,27,28. The HR was 1.69 was similar to that described by Maisey, et al. in a cohort of patients with inoperable pancreatic cancer 21. The adverse effect of female gender in LAPC has not been described in other studies and is not believed to reflect a difference in local treatment practices between men and women. Women represented 44% of this study cohort and experienced an inferior OS. A previous study has reported a significant effect of gender with males experiencing an inferior outcome to females, HR 1.52, *P* = 0.02 21.

In the current study, tumor location did not influence duration of survival. A previous retrospective review of 215 patients, the majority of whom had metastatic disease, reported shorter OS for those with tumors located in the pancreatic tail 29. No association between age and OS was noted in the current study. The lack of effect of age on prognosis may be a reflection of the short median OS duration and that all patients included in the study were able to receive palliative chemotherapy. Weight loss and baseline CEA have previously been reported as significant prognostic factors in another retrospective study 30, but data on these factors were not available for subjects in this study.

To the best of our knowledge, this is the first study to report patterns of metastatic disease and prognostic factors in a population-based cohort of LAPC. Results should be interpreted in the context of several limitations. The retrospective, nonrandomized nature of this review relies on accuracy of written records and information captured by them. While baseline serum levels CA19-9 were available for the majority of patients, the exact timing of its measurement was not identified. Although there was no clinical or radiographic evidence of metastatic disease in approximately 40% of the cohort, the presence of metastatic disease at the time of death was not confirmed by autopsy. Finally, the noted prognostic factors may not apply to LAPC patients who are treated with upfront chemoradiotherapy rather than chemotherapy alone.

In conclusion, ECOG PS, baseline CA 19.9, and development of metastatic disease were significant prognostic factors among LAPC treated with palliative chemotherapy. Our results demonstrate distinct prognostic factors relevant to LAPC and justify the separation of LAPC and metastatic PDAC in clinical trials 13. An estimated 40% of patients succumb to tumor complications and a better understanding of molecular factors predictive of locoregional disease is required.
